# Genetic oxidative stress variants and glioma risk in a Chinese population: a hospital-based case–control study

**DOI:** 10.1186/1471-2407-12-617

**Published:** 2012-12-22

**Authors:** Peng Zhao, Lin Zhao, Peng Zou, Ailin Lu, Ning Liu, Wei Yan, Chunsheng Kang, Zhen Fu, Yongping You, Tao Jiang

**Affiliations:** 1 Department of Neurosurgery, the First Affiliated Hospital of Nanjing Medical University, Nanjing, 210029, China; 2 Department of Neurosurgery, Tiantan Hospital, Capital Medical University, Beijing, 100050, China; 3 Department of Neurosurgery, Tianjin Medical University General Hospital, Tianjin, 300052, China

**Keywords:** Oxidative stress, Single nucleotide polymorphism, Glioma, SOD2, SOD3, GPX1, NOS1

## Abstract

**Background:**

The oxidative stress mechanism is of particular interest in the pathogenesis of glioma, given the high rate of oxygen metabolism in the brain. Potential links between polymorphisms of antioxidant genes and glioma risk are currently unknown. We therefore investigated the association between polymorphisms in antioxidant genes and glioma risk.

**Methods:**

We examined 16 single nucleotide polymorphisms (SNPs) of 9 antioxidant genes (*GPX1, CAT, PON1, NQO1, SOD2/MnSOD, SOD3,* and NOS1*2*3) in 384 glioma and 384 control cases in a Chinese hospital-based case–control study. Genotypes were determined using the OpenArray platform, which employs the chip-based Taq-Man genotyping technology. The adjusted odds ratio (OR) and 95% confidence interval (CI) were estimated using unconditional logistic regression.

**Results:**

Using single-locus analysis, we identified four SNPs (*SOD2* V16A*, SOD3* T58A*, GPX1* -46 C/T*,* and *NOS1* 3’-UTR) that were significantly associated with the risk of glioma development. To assess the cumulative effects, we performed a combined unfavourable genotype analysis. Compared with the reference group that exhibited no unfavourable genotypes, the medium- and high-risk groups exhibited a 1.86-fold (95% CI, 1.30-2.67) and a 4.86-fold (95% CI, 1.33-17.71) increased risk of glioma, respectively (*P*-value for the trend < 0.001).

**Conclusions:**

These data suggest that genetic variations in oxidative stress genes might contribute to the aetiology of glioma.

## Background

Glioma is the most common form of primary brain tumour in adults and generally exhibits a poor prognosis [1-3]. According to the Chinese Health Statistics Yearbook, the incidence of glioma is approximately five to ten per 100,000 person-years in China. The incidence rate has steadily increased despite significant advances in the diagnosis and treatment of glioma [4]; this increase might be attributed to improvements in diagnostic imaging technology.

The aetiology of this malignancy remains largely unknown. People with inherited diseases such as Li-Fraumeni disease, Neurofibromatosis type 1, and Turcot’s disease type 1 exhibit a significantly increased risk of glioma, and consistent with this diversity of predisposing genetic backgrounds, large-scale sequencing of the glioblastoma genome has revealed many genetic alterations [5,6]. Furthermore, there have been many relevant studies focused on the role of polymorphism analysis of candidate genes in glioma risk [7-10]. Taken together, the evidence thus far provides us with important insight for our understanding of the aetiology of and susceptibility for gliomas.

In recent years, the oxidative stress response has been of particular interest in gliomas, given the high rate of oxygen metabolism in the brain [11]. An excess of oxidative stress, which is triggered by reactive oxygen species (ROS) or reactive nitrogen species (RNS), appears to increase the predisposition for glioma, as an elevated concentration of ROS/RNS can cause DNA damage, repress the activity of cellular enzymes, influence apoptosis and proliferation, and promote tumourigenesis [12,13].

To prevent and mitigate damage caused by ROS/RNS and to maintain redox homeostasis, aerobic organisms have developed efficient defence systems mediated by enzymatic and non-enzymatic antioxidants that can act in a coordinated network [14]. Enzymatic antioxidant defences include superoxide dismutase (SOD), glutathione peroxidase (GPx), catalase (CAT), paraoxonase (PON), NADPH-quinone reductase (NQO), and nitric oxide synthase (NOS). Intrinsic antioxidant enzymes are vital to the regulation of oxidative stress responses within cells. Genetic variation in these genes might impact the elimination of ROS/RNS and hence increase cancer risk through ROS/RNS effects [15].

In humans, single nucleotide polymorphisms (SNPs) account for a significant proportion of observed genetic mutations and might be associated with cancer risk by altering the expression levels and functions of the affected genes. Numerous studies [9,16-24] have investigated the association between SNPs in enzymatic antioxidant genes and cancer risk in cancers, including breast cancer, prostate cancer, and a small number of gliomas. To examine whether genetic variation in antioxidant genes is linked with glioma susceptibility, we analysed a set of SNPs and assessed their association with the risk of glioma.

## Materials and methods

### Study design and population

The study population consisted of a consecutive series of glioma patients admitted at two centres, specifically, the Department of Neurosurgery of Jiangsu Province Hospital (the First Affiliated Hospital of Nanjing Medical University) and the Chinese Glioma Genome Atlas (Beijing Tiantan Hospital Neurosurgery Centre), from 2005 to 2010. The inclusion criteria for these cases necessitated a newly diagnosed (pathologically or histologically) intracranial glioma (International Classification of Diseases for Oncology, 9th Edition, codes 9380–9481). Histological diagnosis and grading of the tumours were performed in compliance with WHO criteria (World Health Organization, 2007). There were no gender, ethnicity, or cancer stage restrictions on recruitment. After excluding patients with prior cancer history during the baseline visit, a total of 447 glioma patients were invited to participate in the study, of whom 408 (91%) patients consented. Healthy control subjects without a history of cancer were recruited from the health examination clinics of the same two hospitals during the same time period.

The controls matched the case distribution for frequencies of age, sex, ethnicity, and smoking status. Four hundred controls were successfully enrolled. Each participant or proxy was asked to read and sign informed consent agreements in accordance with the requirements of the institutional review board of each participating institution. Following initial patient drop out, 384 glioma patients and 384 cancer-free control patients were included in the final analysis. The study was approved by the Ethics Review Board of Nanjing Medical University.

### Data collection

For both the cases and the controls, information on demographic characteristics, education, occupation, marital status, personal history, family history of cancer in first- and second-degree relatives, and lifestyle habits, including smoking and alcohol consumption, was collected by trained interviewers using a structured questionnaire. Venous blood was collected at each study centre from participants and frozen at −70°C for further molecular analysis.

### Selection of genes and polymorphisms

Through an extensive mining of the databases of the International HapMap Project (HapMap Data Rel 24/phaseII Nov08) and dbSNP, we identified 16 potential functional polymorphisms, which were located within the 5^′^-UTR, 3^′^-UTR, promoter, coding sequence, and splice sites of nine crucial genes involved in oxidative stress response. All of these SNPs exhibit a reported minor allele frequency (MAF) > 0.05 in the general Han Chinese population (Table 1).

**Table 1:** Primary information for 16 genotyped SNPs in oxidative pathway genes

**Genotyped SNPs**	**Location/or Amino acid change**	**MAF for Chinese in database**^**a**^	***P *value for HWE test**^**b**^	**% Genotyping rate**
*GPX1*: rs1800668 C>T	Promoter	0.078	0.706	99.1
*CAT*: rs769214 G>A	5’region	0.293	0.275	97.9
*CAT*: rs7943316 A>T	5’region	0.256	0.822	98.0
*PON1*: rs854552 T>C	3' UTR	0.179	0.195	99.1
*PON1*: rs662 G>A	nsSNP/Q192R	0.430	0.424	97.7
*NQO1*: rs10517 C>T	3' UTR	0.381	0.397	97.7
*NQO1*: rs1800566 T>C	nsSNP/S1P	0.478	0.757	97.5
*MnSOD*: rs4880 T>C	nsSNP/V16A	0.146	0.914	98.8
*MnSOD*: rs5746136 G>A	3' UTR	0.422	0.264	98.7
*SOD3*: rs2536512 G>A	nsSNP/T58A	0.100	0.730	98.6
*SOD3*: rs2695232 C>T	3' UTR	0.367	0.284	98.6
*NOS1*: rs2682826 C>T	3' UTR	0.256	0.163	98.0
*NOS1*: rs1047735 T>C	nsSNP/Q902H	0.488	0.522	98.4
*NOS2*: rs2297518 G>A	nsSNP/L608S	0.175	0.426	97.2
*NOS2*: rs10459953 G>C	Promoter	0.489	0.790	99.1
*NOS3*: rs1799983 G>T	nsSNP/D298E	0.111	0.586	97.9

Abbreviations: *MAF* minor allele frequency, *HWE* Hardy-Weinberg equilibrium.

^a^Minor allele frequency in the Chinese (CHB, Han Chinese in Beijing, China) population, as reported in dbSNP database.

^b^*P* values were calculated from our control genotype.

### Genotyping

Genomic DNA was isolated from peripheral blood leukocytes using phenol-chloroform extraction and proteinase K digestion. Genotyping was performed using the OpenArray platform (Applied Biosystems, Foster City, CA, USA), which employs a chip-based Taq-Man genotyping technology. Genotype calls were made by OpenArray SNP Genotyping Analysis Software version 1.0.3.; laboratory personnel were blinded to the case–control status of each patient sample. For quality control, ten per cent of randomly selected samples were reanalysed with 100% concordant results, and the genotyping success rate for the sixteen SNPs ranged from 97.2% to 99.1%. To further confirm the genotyping results, selected PCR-amplified DNA samples (n = 2, for each genotype) were genotyped a second time using a direct sequencing method, and the results were also consistent.

### Statistical analysis

All statistical analyses were performed with STATA version 10.0 (Stata Corporation, College Station, TX, USA). The Pearson Chi-squared test was used to assess differences between the cases and the controls with regard to categorical variables, such as gender and smoking status, and to compare observed SNP genotype frequencies with those expected under Hardy-Weinberg equilibrium conditions. Student’s *t*-test was used to test for continuous variables, including age and pack-years. Using unconditional logistic regression, we derived odds ratios (ORs) and confidence intervals (CIs) for each polymorphism and associated P-value. Adjusted P-values factored for variables such as age, gender, smoking status, and pack-years were calculated as confounders to exclude potential bias. A test of linear trend with the score was conducted for each SNP using three-level ordinal variable analysis. To correct for multiple comparison testing, we applied the false discovery rate (FDR) [25] method to the *P-*values to reduce the potential for inaccurate findings. In addition to single SNP analysis, we also analysed the association between the total number of unfavourable genotypes and glioma risk. The unfavourable genotypes were combined and categorised according to the tertiles (low, medium, and high risk) of the number of unfavourable genotypes observed in the controls. Using the low-risk group as a reference, we calculated the ORs and 95% CIs for the other subgroups using multivariate logistic regression adjusted for age, gender, smoking status, and pack-years. A two-tailed *P*-value of less than 0.05 was considered statistically significant.

## Results

### Subject characteristics

The distribution of data on age, gender and smoking status for the cases and controls is shown in Table 2. The cases and controls were similar in age, gender, and smoking status. We included a total of 384 cases and 384 controls. Table 1 shows the primary information for 16 genotyped SNPs in oxidative pathway genes, including the location, minor allelic frequencies (MAF), and Hardy-Weinberg equilibrium (HWE) tests for the 16 SNPs and their genotyping rates. The genotype distributions in the cases and controls of all SNPs were consistent with Hardy-Weinberg equilibrium.

**Table 2:** Distribution of selected host characteristics by case–control status in Chinese

**Variables**	**Case (n = 384)**	**Control (n = 384)**	P*
Age, y (mean ± SD)	62.4 ± 10.8	61.5 ± 12.1	0.277
Gender, no. (%)			
Male	222 (57.8)	217 (56.5)	0.715
Female	162 (42.2)	167 (43.5)	
Smoking status, no. (%)			
Never	228 (59.4)	218 (56.8)	0.738
Former	70 (18.2)	72 (18.7)	
Current	86 (22.4)	94 (24.5)	
Pack-years (mean ± SD)	32.7 ± 25.1	30.3 ± 27.7	0.209
Tumor grade, no. (%)			
I	41 (10.7)	0	
II	176 (45.8)	0	
III	86 (22.4)	0	
IV	81 (21.1)	0	

*P values were derived from the ***χ***^2^ test for categorical variables (gender and smoking status) and *t* test for continuous variables (age and pack-years).

### Main analyses of effects due to individual polymorphisms

As shown in Table 3, four SNPs (*SOD2* V16A*, SOD3* T58A*, GPX1* -46 C/T*,* and *NOS1* 3’-UTR) demonstrated a significant association with glioma risk, as determined by the dominant model (variant-containing genotypes versus common homozygote). Compared to *SOD2* 16Val homozygotes, carriers with the *SOD2* 16Ala allele exhibited a more than 1.86-fold increased risk of glioma occurrence (adjusted OR = 1.86; 95% CI = 1.35-2.55), where the risk increased significantly with the increasing number of variant alleles (*P-trend* < 0.001). Similarly, individuals with the SOD3 58A allele exhibited a significant association with the risk of glioma occurrence compared to the 58T homozygotes (adjusted OR = 1.64; 95% CI = 1.20–2.23; *P-trend* < 0.001). Furthermore, we observed an increased risk of glioma occurrence associated with the *GPX1* rs1800668 variant (adjusted OR = 1.18; 95% CI = 0.82–1.69). In contrast, we observed a decreased glioma risk associated with the *NOS1* rs2682826 variant (adjusted OR = 0.61; 95% CI = 0.45-0.82; *P-trend* = 0.017).

**Table 3:** Allelic and genotypic frequencies and risks for glioma in Chinese

**Genotyped SNPs**		**MAF**	**Common homozygote (*n*)**	**Heterozygote (*n)***	**Rare homozygote (*n*)**	**Heterozygote and rare Homozygote (*n*)**	***P *for trend**
***GPX1 *promoter (rs1800668)**	Case	0.12	301	66	13	79	
	Control	0.09	314	64	4	68	0.110
	OR (95% CI)*		Reference	1.05 (0.72-1.53)	**3.30 (1.07-10.24)**	1.18 (0.82-1.69)	
*CAT* 5’region (rs769214)	Case	0.28	198	146	32	178	
	Control	0.29	192	146	36	182	0.609
	OR (95% CI)*		Reference	0.93 (0.69- 1.26)	0.83 (0.49- 1.39)	0.91 (0.68- 1.21)	
*CAT* 5’region (rs7943316)	Case	0.33	180	140	53	193	
	Control	0.29	195	153	32	185	0.074
	OR (95% CI)*		Reference	1.04 (0.77- 1.41)	1.16 (0.75- 1.79)	1.19 (0.89- 1.58)	
*PON1* 3' UTR (rs854552)	Case	0.22	237	120	23	143	
	Control	0.23	231	126	24	150	0.653
	OR (95% CI)*		Reference	0.96 (0.70- 1.30)	0.96 (0.53- 1.75)	0.96 (0.71- 1.28)	
*PON1* Q192R (rs662)	Case	0.35	161	158	52	210	
	Control	0.36	159	167	52	219	0.833
	OR (95% CI)*		Reference	0.98 (0.72- 1.34)	1.06 (0.68- 1.65)	1.00 (0.75- 1.33)	
*NQO1* 3' UTR (rs10517)	Case	0.39	142	170	60	230	
	Control	0.35	154	181	44	225	0.162
	OR (95% CI)*		Reference	0.99 (0.73- 1.35)	1.44 (0.92- 2.26)	1.08 (0.81- 1.45)	
*NQO1* S1P (rs1800566)	Case	0.46	109	181	78	259	
	Control	0.47	108	187	86	273	0.610
	OR (95% CI)*		Reference	1.00 (0.71- 1.39)	0.93 (0.62- 1.40)	0.98 (0.71- 1.34)	
***MnSOD *V16A (rs4880)**	Case	0.22	241	107	31	138	
	Control	0.12	293	81	6	87	**<0.001**
	OR (95% CI)*		Reference	**1.55 (1.11- 2.16)**	**6.05 (2.48- 14.74)**	**1.86 (1.35- 2.55)**	
*MnSOD* 3' UTR (rs5746136)	Case	0.51	95	182	100	282	
	Control	0.46	118	178	85	263	0.058
	OR (95% CI)*		Reference	1.17 (0.83- 1.64)	1.34 (0.90- 1.99)	1.22 (0.89- 1.68)	
***SOD3 *T58A (rs2536512)**	Case	0.25	235	96	44	140	
	Control	0.11	283	73	25	98	**<0.001**
	OR (95% CI)*		Reference	**1.51 (1.06- 2.14)**	**2.01 (1.20- 3.39)**	**1.64 (1.20- 2.23)**	
*SOD3* 3' UTR (rs2695232)	Case	0.41	142	165	75	240	
	Control	0.41	134	172	70	242	0.922
	OR (95% CI)*		Reference	0.87 (0.63- 1.19)	0.97 (0.65- 1.45)	0.90 (0.67- 1.20)	
***NOS1*** 3' UTR **(rs2682826)**	Case	0.25	209	104	23	127	
	Control	0.28	193	164	24	188	**0.017**
	OR (95% CI)*		Reference	**0.57 (0.42- 0.79)**	0.87 (0.47- 1.59)	**0.61 (0.45- 0.82)**	
*NOS1* Q902H (rs1047735)	Case	0.46	123	165	90	255	
	Control	0.49	101	183	94	277	0.197
	OR (95% CI)*		Reference	0.80 (0.57- 1.11)	0.85 (0.57- 1.25)	0.81 (0.60- 1.11)	
*NOS2* L608S (rs2297518)	Case	0.19	241	120	8	128	
	Control	0.17	255	113	9	122	0.584
	OR (95% CI)*		Reference	1.10 (0.80- 1.50)	0.92 (0.35- 2.42)	1.08 (0.80- 1.47)	
*NOS2* promoter (rs10459953)	Case	0.40	140	178	64	242	
	Control	0.44	122	184	73	257	0.173
	OR (95% CI)*		Reference	0.88 (0.64- 1.21)	0.80 (0.53- 1.21)	0.86 (0.64- 1.16)	
*NOS3* D298E (rs1799983)	Case	0.15	282	77	17	94	
	Control	0.12	278	84	15	99	0.856
	OR (95% CI)*		Reference	0.91 (0.64- 1.29)	1.12 (0.55- 2.29)	0.94 (0.68- 1.30)	

*Multivariable adjustment by age, gender (male or female), smoking status (never, former or current), and pack-years.

Data in *boldface* represent *P* < 0.05.

MAF: Minor Allele frequency.

### Combined effects of the unfavourable genotypes

To understand the cumulative effects of these variants on glioma risk, we performed an unfavourable genotype analysis for four SNPs that had significant and borderline significant associations with glioma risk, including rs1800668 (CC), rs4880 (TT), rs2536512 (GG), and rs2682826 (TC+ TT). Compared to the reference group exhibiting no unfavourable genotypes, the OR for the medium risk group with two unfavourable genotypes was 1.86 (95% CI, 1.30-2.76), and the OR was increased to 4.86 (95% CI, 1.33–17.71) for the high-risk group with 3 unfavourable genotypes (Table 4).

**Table 4:** Joint effects of unfavorable genotypes in case patients and control subjects in Chinese

**Risk group (no. unfavorable genotypes)**	**Cases**	**Controls**	OR (95% CI)*
Reference group^a^ (n = 0)	147	205	Reference
Low-risk reference group (n = 1)	96	83	1.51 (1.05- 2.17)
Medium-risk group (n = 2)	108	76	1.86 (1.30-2.67)
High-risk group ^b^ (n ≥3)	11	3	4.86 (1.33-17.71)
*P* for trend			<0.001

*Adjusted for age, gender, smoking status, and pack-years.

^a^Reference group: *GPX1* promoter: CC, *MnSOD* V16A: TT, *SOD3* T58A: GG, and *NOS1* 3' UTR: CT+TT.

^b^Because the subject number in group ‘4’ was sparse (one control and three patients), the subjects with greater than three unfavourable genotypes were combined as the high-risk group.

## Discussion and conclusion

Emerging evidence from in vitro, animal, and human studies has indicated that ROS/RNS and the activation of redox-sensitive signalling pathways play a crucial role in cancer development [26-29]. Such antioxidant mechanisms are extremely important, as they represent the direct removal of ROS/RNS, particularly during gliomatous carcinogenesis. To investigate the potential association between SNPs in antioxidant defence genes and the risk of glioma occurrence, we conducted this case–control study. In this study, we observed a statistically significant association between four SNPs (*SOD2* V16A*, SOD3* T58A*, GPX1* -46 C/T*,* and *NOS1* 3’-UTR) of antioxidant genes and the risk of glioma occurrence in a Chinese population. Additionally, three SNPs exhibited statistically significant evidence of differential dose–response associations. To the best of our knowledge, this is the first report of an association study between antioxidant gene SNPs and glioma risk in a Chinese population.

SODs are a ubiquitous family and represent the most important line of antioxidant enzyme defence against ROS, particularly the superoxide anion radicals [13]. SOD enzymes, which catalyse the spontaneous dismutation of the superoxide radical into hydrogen peroxide, are present in all subcellular milieus of the nervous system, including the mitochondrial intermembrane space (SOD1; copper/zinc SOD); the mitochondrial matrix (SOD2; manganese SOD); and the plasma, lymph, and synovial fluids (SOD3; extracellular SOD) [30]. Superoxide dismutase 2 (SOD2) (also known as manganese superoxide dismutase [MnSOD]) is an essential defender against mitochondrial superoxide radicals.

SOD2 converts the superoxide anion radical into hydrogen peroxide and oxygen within mitochondria and plays a key role in protecting cells from oxidative damage [31]. In the early stages of carcinogenesis, oxidative stress and relatively low levels of MnSOD result in DNA damage and cell injury [32-34]. MnSOD plays a critical role in the defence against oxidant-induced injury and apoptosis of rapidly growing cancer cells, and the tumour-suppressive effects of MnSOD have been well established [12,14,35]. Whereas Chung-man et al. [36] and Izutani et al. [37] previously found increased SOD2 levels in cancer cells, other studies have reported elevated MnSOD expression levels in aggressive cancers compared to benign counterparts, and this increased expression has been proposed to enhance metastasis following cancer progression, possibly through increased expression of matrix metalloproteinases (MMP) [38,39], which is one possible mechanism supporting the role of SOD2 in cancer invasiveness and metastatic capacity. The overexpression of SOD2 can also induce increased levels of hydrogen peroxide (H2O2) [40,41]. H2O2 is a major intracellular oxidant and induces DNA damage in glioma cells [42,43]. Although it might be difficult to determine the precise mechanisms that are most relevant to the pathologies of the patients in this study, the identification of these two possible mechanisms is consistent with our results.

To our knowledge, most epidemiological studies have indicated that SOD2 polymorphisms are linked to clinically significant increases in colon, gastric, lung, breast, and prostate cancers [16-20]. These polymorphisms have also been linked to the development of meningiomas and glioblastomas [44]. Here, our results revealed a statistically significant association between SOD2 rs4880 and the risk of glioma. Rajaraman P et al. [30] showed an increased risk of acoustic neuroma with the SOD2 (Val16Ala) Ala variant, but no significant association between the C genotype and the risk of glioma was observed. The T-to-C nucleotide polymorphism (rs4880), which converts a valine to an alanine in the mitochondrial targeting sequence at position 16 of the protein (Val16Ala), is considered one of the most interesting polymorphisms in the SOD2 gene. The Val-to-Ala transition alters the secondary structure of the protein, resulting in more efficient transport of SOD2 into the mitochondrial matrix. Thus, the C allele can increase the ability of SOD2 to neutralise superoxide radicals compared to the T allele [45,46]. Diffuse invasion into the surrounding brain is a characteristic feature of gliomas, essentially preventing surgical cure, leading to recurrence and representing perhaps the largest obstacle to effective therapy. The invasive nature of glioma cells into the brain parenchyma is intimately linked to the degradation of the extracellular matrix. Activated MMPs are a prerequisite for cancer cell invasion and metastasis. Several lines of evidence have suggested that the overexpression of SOD2 induces a profound increase in the expression of MMP-1 [47-49]. Because the Ala mutant confers a 40% higher MnSOD activity than the Val wild-type form, the increased levels of SOD2 result in increased risk for more invasive glioma activity by inducing MMPs. Our results are consistent with this function of the SOD2 rs4880 in glioma and warrant further investigation. A recent study has indicated that SOD2 rs4880 might significantly modulate the prognosis of breast cancer patients [31], implicating SOD2 rs4880 as a potential prognostic biomarker in gliomas.

Our results also indicate a role for SOD3 rs2536512 in the risk of glioma and demonstrated that the SOD3 A genotype correlated with a significantly increased risk of glioma occurrence in a Chinese population. SOD3 was first detected in human plasma, lymph, ascites, and cerebrospinal fluids [50]. This SNP (rs2536512) results in a threonine-to-alanine conversion that replaces a polar hydrophilic amino acid with an aliphatic hydrophobic amino acid at position 58 of the SOD3 protein, eliminating a PKC delta phosphorylation motif [51]. Few studies have been performed to examine the association between SOD3 rs699473 and glioma risk or to explore the association between SOD3 rs2536512 and cerebral infarction in women [52]. Our study has demonstrated an association between the human SOD3 gene and the risk of glioma occurrence. Additionally, we observed statistically significant evidence that carriers of the SOD2 and SOD3 variants exhibit increased glioma dose–response relationships compared with homozygous wild-type subjects (P-trend < 0.001). Confirmation of our findings in alternate populations represents a high priority. The SOD SNP-associated glioma risks observed in our study suggest that the amino acid changes caused by these SNPs might be physiologically significant in the development of cancer.

GPX1 encodes the antioxidant glutathione peroxidase isoform 1 and acts in conjunction with the tripeptide glutathione (GSH), which is present in cells in high (micromolar) concentrations [53]. Accumulating data link altered or abnormal GPX1 expression with the aetiology of cancer [54-56]. The additional identification of GPX1 polymorphisms, concordant with several other studies, suggests the involvement of GPX1 variants in the aetiology of glioma [30,57]. In these previous studies, the effect sizes occurred at an odds ratio of approximately 1.1; in our study, the rs1800668 SNP in GPX1 was associated with an almost 3.3-fold increased risk when rare homozygotes were compared to common homozygotes. Although the previous studies indicated the same association that was observed in our results, they lacked statistical significance and association. Thus, it is likely that some associations that we have presented here are chance findings. These data only provide evidence that GPX1 rs1800668 contributes to glioma predisposition. Further epidemiologic and functional studies in a larger population are warranted to validate these results.

Nitric oxide (NO), a pleiotropic messenger molecule, is predominantly produced from the precursor L-arginine by neuronal nitric oxide synthase (NOS1) in the central nervous system [58,59]. The possible involvement of NOS1 rs2682826 in vital functions has been suggested by several studies. The rs2682826 SNP is located in the 3^′^-UTR of exon 29 of NOS1 gene. It has been established that the 3^′^-UTR plays a role in the stability and translational efficiency of the mRNA transcript [60]. Additionally, the rs2682826 SNP is proximally located to several miRNA-binding sites within the gene’s 3^′^-UTR. Differences in protein translation might occur depending on the presence of the SNP in the mRNA of this gene [24]. Further functional analyses are required to clarify these possibilities.

In this population sample, NOS1 rs2682826 might play a protective role in the development of glioma under the dominant model (adjusted OR = 0.61; 95% CI = 0.45–0.82; *P-trend* = 0.017). Additional evidence substantiating the physiological relevance of the NOS1 rs2682826 polymorphisms was previously revealed by Ibarrola-Villava et al. [24], who found that NOS1 rs2682826 is associated with protective effects in malignant melanoma, accounting for a 40% reduction. If confirmed, the evidence presented in this study here would facilitate the identification of individuals who possess the heterozygote or rare homozygote marker of NOS1 rs2682826. These patients would particularly benefit from glioma treatments.

However, the limitations of our study must be addressed. First, these findings cannot be generalised to other populations because our study was specifically conducted using a Chinese population. Second, the number of cases and controls included in this study was relatively small; thus, further studies with larger sample-sizes are needed.

In conclusion, we have demonstrated that the influence of these genetic variations in the oxidative response has a potential regulatory effect on glioma tumourigenesis, and furthermore, we have identified a trend towards an increasing glioma risk associated with an increasing number of unfavourable genotypes that occur in a dose-dependent manner. To our knowledge, this study provides the first epidemiological evidence that supports an association between oxidative response-related genes and glioma risk in a Chinese population. Further studies are warranted to assess the observed effects using a more comprehensive collection of SNPs in oxidative response genes.

## Abbreviations

SOD: Superoxide dismutase; GPx: Glutathione peroxidase; CAT: Catalase; PON: Paraoxonase; NQO: NADPH-quinone reductase; NOS: Nitric oxide synthase; SNP: Single nucleotide polymorphism; MMP: Matrix metalloproteinases; OR: Odds ratio; CI: Confidence interval.

## Competing interests

The authors declare that they have no competing interests.

## Authors‘ contributions

PZ and LZ participated in the collection of data and manuscript preparation. PZ and LZ performed the statistical analysis. NL, WY, CK, ZF, YY and TJ collected the samples. PZ and AL participated in the study design and critically revised the manuscript. PZ and TJ participated in the study design and manuscript preparation. All of the authors read and approved the final manuscript.

## Pre-publication history

The pre-publication history for this paper can be accessed here:

http://www.biomedcentral.com/1471-2407/12/617/prepub
